# Adjunctive use of systematic retinal thickness map analysis to monitor disease activity in punctate inner choroidopathy

**DOI:** 10.1186/s12348-016-0073-4

**Published:** 2016-03-10

**Authors:** Savitha Madhusudhan, Pearse A. Keane, Alastair K. Denniston

**Affiliations:** Centre for Rare Diseases, University Hospitals Birmingham NHS Foundation Trust, Birmingham, UK; Ophthalmology Department, University Hospitals Birmingham NHS Foundation Trust, Birmingham, UK; NIHR Biomedical Research Centre for Ophthalmology, Moorfields Eye Hospital NHS Foundation Trust and UCL Institute of Ophthalmology, London, UK; Centre for Translational Inflammation Research, Institute of Inflammation and Ageing, College of Medical and Dental Sciences, University of Birmingham, Birmingham, UK

**Keywords:** Punctate inner choroidopathy, Retinal thickness map, White dot syndromes, SD-OCT, Macular grid

## Abstract

A challenge in the management of ‘white dot syndromes’ is the lack of sensitive objective measures of disease activity. Retinal thickness maps from spectral domain optical coherence tomography (SD-OCT) inform treatment decisions in other retinal conditions such as age-related macular degeneration and diabetic maculopathy. In this report, we demonstrate their value in providing quantitative monitoring of a patient with punctate inner choroidopathy (PIC). Retinal thickness maps referenced against a baseline scan reliably detected focal areas of increased macular volume in active PIC lesions during symptomatic episodes, highlighting these as ‘hot spots’ that could be quantified, providing an objective basis for treatment decisions.

## Findings

### Introduction

Punctate inner choroidopathy (PIC) is a rare idiopathic inflammatory condition affecting the outer retina, retinal pigment epithelium and choroid that mostly occurs in young myopic females and predominantly affects the posterior pole [1]. It runs a variable course, with multiple recurrences in up to a third of patients and can be associated with severe visual loss when complicated by choroidal neovascularisation (CNV) and subretinal fibrosis [2]. Stages in the evolution of PIC lesions on spectral domain optical coherence tomography (SD-OCT) have been described [3, 4]. Although blue wavelength fundus autofluorescence (FAF) can help discriminate between active and atrophic lesions, findings can sometimes be ambiguous [5]. We describe the application of systematic retinal thickness map analysis of SD-OCT (Heidelberg Eye Explorer™, Heidelberg, Germany) as a useful adjunct in detecting lesion activity in PIC.

### Case example

A 43-year-old Caucasian female was diagnosed with PIC after presenting with photopsia associated with active retinal lesions and multifocal chorioretinal atrophic lesions in the macula in her left eye. Her right eye had long-standing macular scarring with vision of ‘hand movements’. Oral corticosteroid treatment for her active PIC lesions resulted in visual acuity improving from 6/12 to 6/6 in the left eye. It was noted that although treatment response was not easily determined on non-quantitative assessment of the SD-OCT images, reduction in lesion size could easily be detected and objectively measured using retinal thickness maps (Heyex™).

**Fig. 1:**
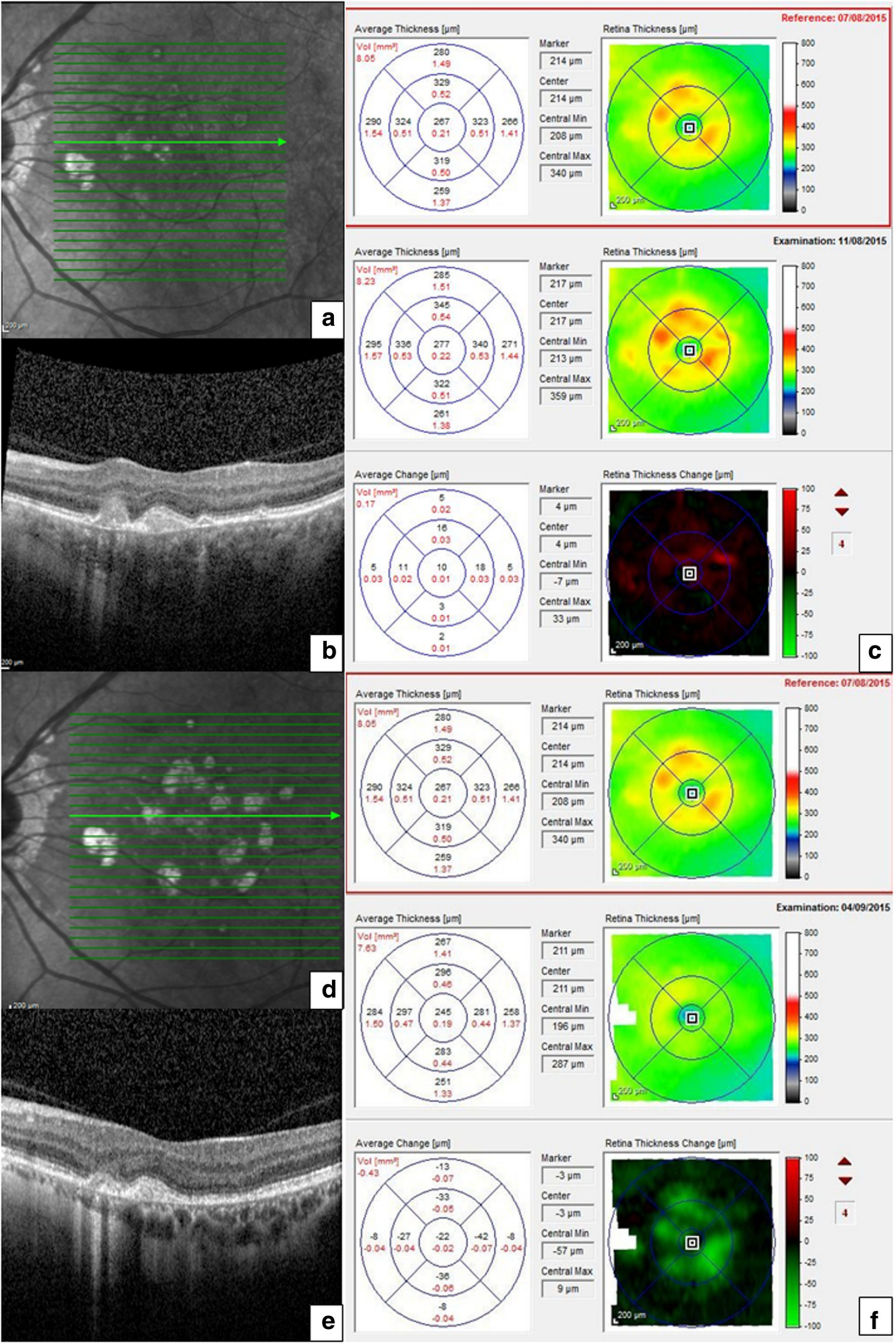
Comparative retinal thickness maps and SD-OCT images from an active and subsequent quiescent phase of punctate inner choroidopathy lesions in the patient’s left eye. SD-OCT during a symptomatic flare-up showing focal disruption of the RPE and ellipsoid zone associated with a dome-shaped hyperreflective area (**a**, **b**) and corresponding retinal thickness maps showing an increase in retinal thickness in the form of increase in the number, size and intensity of ‘hot spots’ compared to the reference with quantitative analysis of average change (**c**) in different subfields of the macular grid. SD-OCT was repeated 23 days after intravitreal triamcinolone for the above episode showing involution of the active lesion (**d**, **e**) and resolution of the hot spots on the corresponding retinal thickness maps compared to the same reference scan, together with quantitative analysis of the average change in retinal thickness (**f**)

Recurrent exacerbations of the disease resulted in increasing maintenance treatment (mycophenolate mofetil 1 g bid; oral prednisolone 10 mg od) with individual flares being treated with intravitreal corticosteroid; a choroidal neovascular membrane was treated with a course of intravitreal bevacizumab injections. Retinal thickness maps based on automated segmentation lines delineating internal limiting membrane and Bruch’s membrane proved consistently reliable, showing focal retinal thickening in the form of ‘hot spots’ at sites which were suspicious for new activity on SD-OCT (Table 1). Following intravitreal steroid treatment, the thickness map returned towards baseline each time, corresponding with the resolution of symptoms (Fig. 1).

**Table 1:** Outcomes of methods used to detect active PIC lesions in the patient’s left eye

Symptom episodes	Unaided Snellen visual acuity (pinhole vision when tested)	Clinically identifiable lesion	New changes in FAF	New changes in SD-OCT	‘Hot spots’ on thickness map	Maximal increase in thickness within a ‘hot spot’ compared to immediate previous visit (μm)	Treatment
1 December 2014	6/6	No	No	Yes	Yes	56	IVTA
27 January 2015	6/7.5	Yes	No	Yes	Yes	102	IVTA
13 March 2015	6/9 (6/6)	Doubtful	FAF unavailable	Yes	Yes	103	IVTA
12 May 2015	6/6	No	No	Yes	Yes	12	Intravitreal dexamethasone implant
11 August 2015	6/9	No	FAF unavailable	Yes	Yes	21	IVTA
29 September 2015	6/9	No	Doubtful	Yes	Yes	67	Intravitreal dexamethasone implant

*FAF* fundus autofluorescence, *IVTA* intravitreal triamcinolone

These maps also enabled an estimation of what constituted a ‘symptomatic’ deterioration. Comparing each ‘active’ episode to the preceding ‘quiescent’ visit showed that the mean detectable change of retinal thickness associated with symptoms was 60.2 μm (range 12–103 μm). The average increase in retinal thickness during a flare in the inner (1 mm), middle (3 mm) and outer (6 mm) zones of the ETDRS macular grid was 18.2 μm (range 10–33 μm), 25.9 μm (range 3–59 μm) and 12.6 μm (range 14–28 μm), respectively, with more hot spots recurring in the middle, inner and outer zones in that order (Table 1). This patient has given specific consent for her clinical data and images to be published.

### Discussion

A major challenge in managing patients with ‘white dot syndromes’ is the lack of sensitive measures of disease activity that can provide objective guidance for treatment decisions. This is particularly important when dealing with sight-threatening conditions where systemic treatment with immunosuppressants may be indicated, such as PIC.

Our patient was highly sensitive to visual symptoms in her ‘only’ eye, but similar changes may often be missed by patients (e.g. if occurring in the worse-seeing eye). For the clinician, the changes in PIC may be subtle and may be missed on clinical examination or non-quantitative assessment of imaging. In our dedicated PIC clinic, all patients have longitudinally tracked retinal thickness maps facilitating detection of new lesions even in asymptomatic patients, guiding treatment decisions and quantifying treatment response. This case highlights how changes on these maps can easily be visualised, providing an objective correlate to the patient’s symptoms and enabling confidence in treatment. The use of these thickness maps enables rapid screening for new or changing lesions which can then be analysed in more detail on the ‘b mode’ SD-OCT scans; thickness maps therefore augment rather than replace the ‘b scans’.

This technique depends on accurate segmentation of the SD-OCT images, which can be readily checked prior to interpretation of maps. Extensive chorioretinal scarring may cause failure of automated segmentation but can be easily overcome by applying manual segmentation. Decentration of the macular grid will also affect individual subfield thickness measurements, so all ‘follow-up’ images should be acquired or interpreted against a designated reference scan [6]. It should be noted that the technique is not specific to the Heyex™ software. Most OCT manufacturers provide retinal thickness maps as standard, and therefore, this application should be widely available for monitoring patients with PIC or other white dot syndromes. Devices do, however, vary in their resolution and in the accuracy of their registration between baseline and follow-up scans. This may affect the reliability of detecting change over time, particularly the subtle but functionally significant changes seen in many white dot syndromes.

In PIC, recurring inflammatory lesions occurring close to fixation may lead to a significant loss of vision and immunomodulatory treatment is advised. Additionally, the clinician should be alert to the development of inflammatory CNV, which is a relatively common complication of PIC, and should be confirmed by retinal angiography and timely treatment initiated including the use of anti-vascular endothelial growth factor (anti-VEGF) therapy [7]. There is little data to support the use of one immunosuppressant over another in PIC, but Neri has reported on the combination of high-dose corticosteroid in combination with mycophenolate mofetil (MMF) for the treatment of refractory inflammatory CNV, including five eyes with PIC. In this series, the regimen of three pulses of 1 g intravenous methylprednisolone, oral prednisolone (starting at 1 mg/kg/day) and MMF (1 g twice daily) was well tolerated and led to stabilisation or improvement in visual acuity and stabilisation or reduction of lesion size in all lesions [8]. SD-OCT is already a valued tool in the objective assessment of macular oedema in uveitis and is increasingly finding new applications [9]. In these instances, the use of retinal thickness maps is helpful to ascertain lesion activity. In conclusion, retinal thickness map tracking provides a sensitive, reliable, objective way of monitoring PIC and, in this era of multimodal imaging, it may prove a useful adjunct in managing other conditions in the spectrum of white dot syndromes.

## Abbreviations

PIC: punctate inner choroidopathy
CNV: choroidal neovascularisation
SD-OCT: spectral domain optical coherence tomography
FAF: fundus autofluorescence
